# Aging hallmarks and innate immune responses in human monocytes and subsequent chronic conditions

**DOI:** 10.1093/geroni/igaf122.3329

**Published:** 2025-12-31

**Authors:** Madeline Kelly, Da Ma, Jingzhong Ding

**Affiliations:** Wake Forest University School of Medicine, Maple Grove, Minnesota, United States; Wake Forest University School of Medicine, Winston-Salem, North Carolina, United States; Wake Forest University School of Medicine, Winston-Salem, North Carolina, United States

## Abstract

Animal studies suggest that the aging process, underlain by interconnected cellular alterations, promote multiple aging-related chronic conditions simultaneously. Translating these animal findings to humans is needed to realize the therapeutic potentials of geroscience. Transcriptomic profiles were determined in circulating monocytes from 1,264 MESA participants aged 55-94 (51% women, 53% minority). Older age was associated with alterations in 24 of 40 co-expression modules (FDR< 0.05), including interconnected (r > 0.4 or r<-0.4) hallmarks of the aging process (such as cellular senescence, mitochondrial dysfunction, loss of proteostasis, and epigenetic alterations) and innate immune responses of monocytes (such as inflammatory responses and complement activation). Innate immune responses were further elevated in persons with obesity (FDR< 0.05). Innate immune responses were cross-sectionally associated with shorter 6 minute walk distances (inflammatory responses and complement activation) and lower Cognitive Abilities Screening Instrument scores (inflammatory responses) (FDR< 0.05). During a 6-year follow-up, complement activation predicted new events of aging-related diseases including cardiovascular disease, type-2 diabetes, hypertension, cancer, dementia, chronic kidney disease, chronic obstructive pulmonary disease, and hip fracture (RH:1.16-1.18. FDR=0.03). In conclusion, our transcriptomic analyses identified a coordinated network of aging-related dysfunctions of cellular hallmarks and innate immune responses in human monocytes, and these innate immune responses, exacerbated by obesity, may contribute to poor physical and cognitive performance and aging-related diseases.

